# Necroptosis regulated proteins expression is an early prognostic biomarker in patient with sepsis: a prospective observational study

**DOI:** 10.18632/oncotarget.21099

**Published:** 2017-09-20

**Authors:** Bing Wang, Jian Li, Hong-Mei Gao, Ying-Hong Xing, Zhu Lin, Hong-Jie Li, Yong-Qiang Wang

**Affiliations:** ^1^ Department of Intensive Care Unit and Key Laboratory for Critical Care Medicine of the Ministry of Health, Emergency Medicine Research Institute, Tianjin First Center Hospital, Tianjin 300192, China

**Keywords:** sepsis, receptor interacting protein kinase 3, receptor interacting protein kinase 1, mortality, biomarker

## Abstract

**Background and aim:**

Increasing researchers indicate that necroptosis is playing an important role in the regulation of systemic inflammatory response syndrome. The current study was to investigate the prognostic biomarker of the regulated proteins of necroptosis in sepsis patients.

**Results:**

One hundred and twenty-four patients were divided into three groups: 43 patients (34.68%) with sepsis, 39 patients (31.45%) with severe sepsis, and 42 patients (33.87%) with septic shock. The RIPK3 levels in the severe sepsis and septic shock groups were notably higher than those in sepsis group at various time points (all *p* < 0.05), and the RIPK3 levels had positive association with the Sequential Organ Failure Assessment (SOFA) score as well as procalcitonin (PCT) level (all *p* < 0.05). The RIPK3 level like the SOFA score and PCT level could be a prognostic biomarker of sepsis patients.

**Materials and Methods:**

We prospectively recruited the eligible patients with sepsis, severe sepsis, or septic shock who were treated in our hospital from January 2014 to May 2016. The dynamic changes in infectious variables and blood plasma receptor interacting protein kinase 1 (RIPK1) and receptor interacting protein kinase 3 (RIPK3) levels were determined from measurements taken in a double-blinded fashion at 24, 48, 72, and 120 hours later.

**Conclusions:**

These results suggested that dynamic monitoring of RIPK3 levels can contribute to the prediction of outcome of sepsis and might be of particular value in identifying patients who would benefit from specific treatments.

## INTRODUCTION

Sepsis is a life-threatening condition that arises when the body's response to infection causes injury to its own tissues and organs. The incidence of sepsis has yet to be determined, however, it is the most cause of mortality worldwide. For the initial phases of sepsis, early detection, appropriate classification, and suitable treatment have shown to be associated with improved patient outcome in intensive care units (ICUs). Indeed, the optimum timing of intervention seems to be vital and indicates the major determinant of survival for patients with sepsis.

In clinical practice, the “gold standard” for diagnosis of sepsis is blood culture. However, it usually has the results delay of about 3 to 7 days, and false negative results occur frequently. In addition, some scoring systems to commonly evaluate organ dysfunction or prognosis in patients with sepsis, such as the Sequential Organ Failure Assessment (SOFA) and the Acute Physiology and Chronic Health Evaluation (APACHE), have not provided sufficient information to determine their accuracy of the prognosis for the severity and mortality of sepsis [1–3].

Recent studies demonstrated that some biomarkers have been investigated for diagnosing and monitoring sepsis, such as C-reactive protein (CRP) and procalcitonin (PCT), however, these biomarkers levels were also detected to be elevated in patients with many noninfectious conditions, for instance, malignant tumors, acute coronary syndromes, severe trauma, burns, rheumatic disorders, or post-surgery, etc. [4–7]. Therefore, a new septic biomarker is needed.

Necrosis has been considered an accidental mode of cell death in cells; however, it has recently been recognized that necroptosis, a programmable form of necrosis, may be regulated via defined signal transduction pathways [8]. For example, receptor interacting protein kinase 3 (RIPK3) and receptor interacting protein kinase 1 (RIPK1) are two critical kinases responsible for mediating necroptosis [9–10]. Further, the mixed lineage kinase domain-like (MLKL) pseudokinase is a direct executioner of necroptosis [11]. Thus, RIPK3, RIPK1, and MLKL activation are currently synonymous with the onset of necroptosis, and increasing researchers indicate that necroptosis is playing an important role in the regulation of systemic inflammatory response syndrome and critical care medicine [12–14].

The usage of necroptosis regulated proteins in monitoring response to therapeutic interventions would guide development of novel treatment strategies to patients with sepsis. In the light of the clinical importance of the early treatment strategy for sepsis prior to the onset of clinical deterioration, the current study was to determine the prognostic biomarker of the regulated proteins of necroptosis in sepsis patients.

## RESULTS

### Characteristics of patients

One hundred and thirty-one patients with sepsis, who were admitted to our medical ICU from January 2014 to May 2016, were enrolled. Seven patients were excluded due to receipt of platelet transfusion, acute gastrointestinal hemorrhage, and insufficient data. Finally 124 eligible patients were categorized by severity into sepsis (*n* = 43, 34.7%), severe sepsis (*n* = 39, 31.5%), and septic shock (*n* = 42, 33.8%) groups.

Among the included sepsis patients, the primary infection sites, the concurrent chronic diseases, and the regulated proteins of necroptosis including RIPK3 and RIPK1 levels were prospectively collected. There were significant differences in the patients’ characteristics of APACHE II score, SOFA score, CRP level, PCT level, and RIPK3 level at 24 hours after sepsis diagnosed among three groups. The patients’ characteristics are shown in Table 1.

**Table 1:** Characteristics and outcomes of patients with sepsis

Variables	All (124 patients)	Sepsis (43 patients)	Severe sepsis (39 patients)	Sepsis shock (42 patients)	*P* values
Sex (female/male)	63/61	21/22	20/19	22/20	0.95
Age, median (range), years	61 (17–81)	59 (17–76)	60 (17–80)	63 (18–81)	0.98
APACHE II score, median (range)	24 (2–45)	22 (3–43)	29 (2–45)	35 (3–45)	0.0001
SOFA score, median (range)	7.4 (0–18.2)	7.0 (0–18.2)	9.2 (0–17.6)	12.1 (1–16.4)	0.001
CRP, mg/dL, median (range)	12.3 (2.8–39.7)	12.5 (3.2–35.8)	14.9 (2.8–34.7)	17.4 (4.2–39.7)	0.01
PCT, ng/mL, median (range)	4.3 (2.1–36.2)	4.1 (3.16–34.2)	6.2 (2.1–35.1)	7.6 (1.2–36.2)	0.04
RIPK3, pg/mL, median (range)	14.7 (1.2–32.5)	7.9 (3.2–16.7)	11.3 (1.2–19.2)	18.9 (3.6–32.5)	0.001
RIPK1, pg/mL, median (range)	8.2 (1.8–25.1)	7.1 (2.7–15.6)	8.4 (1.8–14.5)	8.9 (2.4–25.1)	0.72
Creatinine, mg/dL, median (range)	1.1 (0.2–14)	1.1 (0.4–13.5)	1.0 (0.2–12.9)	1.3 (0.3–14.1)	0.65
WBC, × 10^9^ L, median (range)	11.0 (2.5–32.1)	10.5 (3.1–28)	10.9 (2.8–29.2)	11.7 (2.5–32.1)	0.78
PLT, × 10^3^ uL, median (range)	73 (29–378)	85 (62–158)	78 (57–312)	56 (29–378)	0.23
PT, seconds, mean ± SD	16.73 ± 5.35	14.25 ± 3.4	16.53 ± 3.52	18.63 ± 5.7	0.53
Primary infection site					0.99
Respiratory	70 (56.4%)	21 (48.8%)	23 (58.9%)	26 (61.9%)	
Abdominal	10 (8.1%)	3 (6.9%)	2 (5.1%)	5 (11.9%)	
Urinary tract	18 (14.5%)	5 (11.6%)	4 (10.2%)	9 (21.4%)	
Bloodstream	8 (6.5%)	3 (6.9%)	2 (5.1%)	3 (7.1%)	
Others	13 (10.5%)	4 (9.3%)	3 (7.6%)	6 (14.2%)	
Unknown	5 (4.0%)	1 (2.3%)	2 (5.1%)	2 (4.7%)	
Microorganism identified					0.92
Gram-negative bacilli	58 (46.8%)	21 (48.8%)	20 (51.2%)	17 (40.5%)	
Gram-positive cocci	48 (38.7%)	16 (37.2%)	13 (33.3%)	19 (45.2%)	
Fungus	8 (6.5%)	3 (6.9%)	3 (7.7%)	2 (4.8%)	
Intracellular germs	4 (3.2%)	1 (2.3%)	2 (5.2%)	1 (2.4%)	
Others	6 (4.8%)	2 (4.8%)	1 (2.5%)	3 (7.1%)	
The type of organ dysfunction†				1(2.3%)	1.00
CRF	60 (48.4%)	19 (44.2%)	17 (43.5%)	24 (57.1%)	
CHF	65 (52.4%)	22 (51.2%)	20 (51.3%)	23 (53.5%)	
CKD	41 (33.1%)	13 (30.2%)	12 (30.8%)	16 (38.1%)	
Hepatic insufficiency	20 (16.1%)	6 (13.9%)	6 (15.4%)	8 (19.1%)	
Immune suppression	14 (11.3%)	4 (9.3%)	4 (10.2%)	6 (14.2%)	
Others	5 (4.0%)	2 (4.6%)	1 (2.6%)	2 (4.8%)	
The number of organ dysfunction					0.96
1	35 (28.2%)	13 (30.2%)	11 (28.2%)	11(26.2%)	
2	43 (34.7%)	16 (37.2%)	13 (33.3%)	14 (33.3%)	
≥ 3	46 (37.1%)	14 (32.6%)	15 (38.5%)	17 (40.5%)	
Twenty-eight days mortality					0.03
Alive	49 (39.5%)	22 (51.2%)	17 (43.6%)	10 (23.8%)	
Dead	75 (60.5%)	21 (48.8%)	22 (56.4%)	32 (76.2%)	

†Some of included patients had more than one co-morbidity.

Abbreviations: APACHE II score: Acute Physiology and Chronic Health Evaluation score; SOFA: Sequential Organ Failure Assessment; CRP: C-reactive protein; PCT: procalcitonin; RIPK3: receptor interacting protein kinase 3; RIPK1: receptor interacting protein kinase 1; WBC: white blood cell; PLT: platelet; PT: prothrombin time; CRF: chronic respiratory failure; CHF: chronic heart failure; CKD: chronic kidney dysfunction.

### Changes in SOFA score and RIPK3, RIPK1, CRP, and PCT levels

At 24 hours after sepsis diagnosed, the plasma RIPK3 and RIPK1 levels were 7.9 (3.2–16.7) pg/mL and 7.1 (2.7–15.6) pg/mL for sepsis patients, 11.3 (1.2–19.2) pg/mL and 8.4 (1.8–14.5) pg/mL for severe sepsis patients, as well as 18.9 (3.6–32.5) pg/mL and 8.9 (2.4–25.1) pg/mL for septic shock patients. The RIPK3 levels in severe sepsis and septic shock group were markedly higher than those in sepsis group at various time points (all *p* < 0.05), and trends were remarkably similar for SOFA scores, CRP levels, and PCT among these groups but not RIPK1 levels and WBC counts.

**Figure 1:**
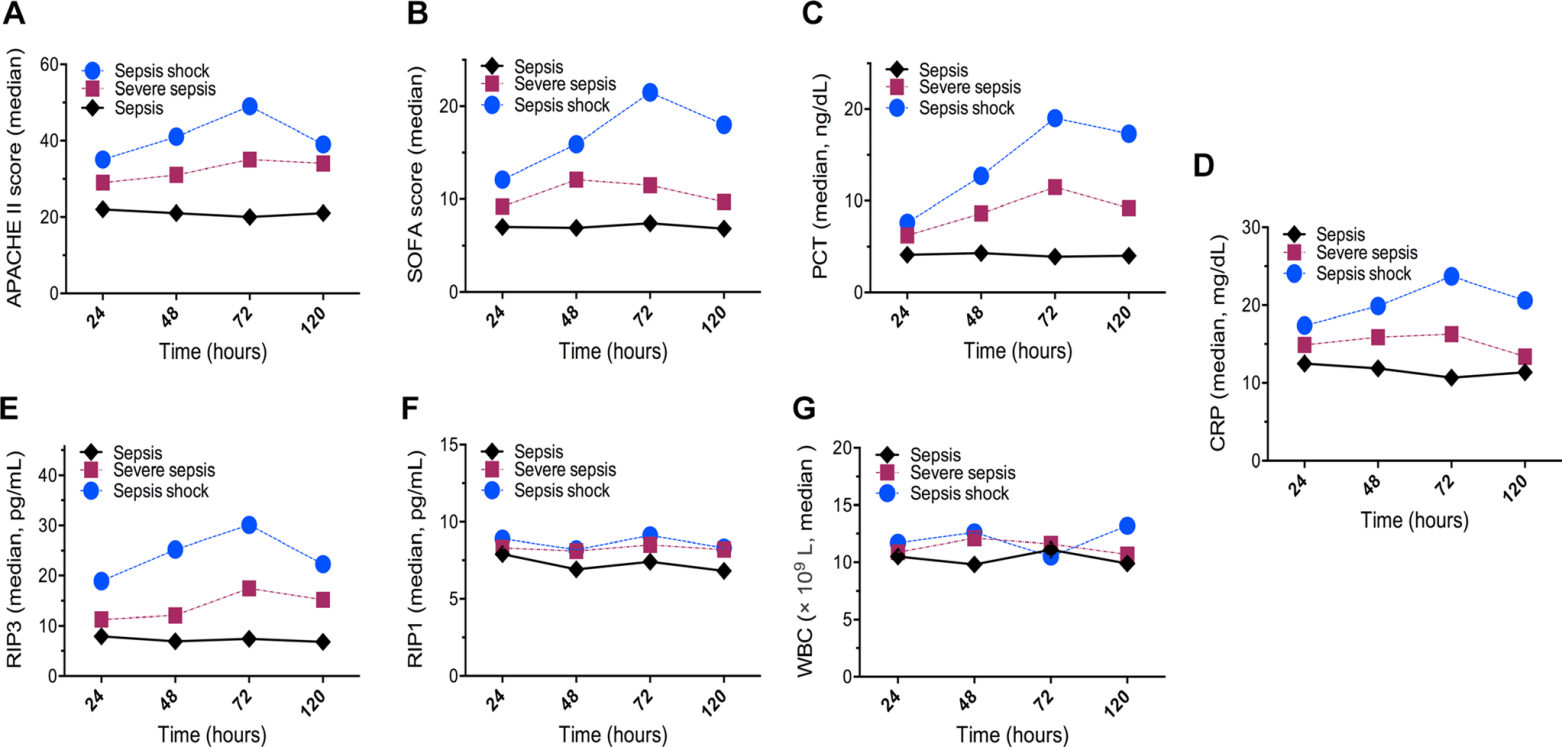
Dynamic changes in the median values of the levels of infectious markers, RIP3, and RIP1 among patients with sepsis, severe sepsis, and septic shock (**A**) APACHE II scores; (**B**) SOFA scores; (**C**) serum PCT levels; (**D**) serum CRP levels; (**E**) plasma RIP3 levels; (**F**) plasma RIP1 levels; and (**G**) WBC counts.

In addition, RIPK3 levels in severe sepsis and septic shock patients peaked at 72 hours and afterwards declined gradually, which might suggest that inflammation in those two groups was at the highest levels by 72 hours. While RIPK3 levels in sepsis patients as well as the RIPK1 levels in sepsis, severe sepsis, and septic shock patients did not remarkably change within 120 hours (Figure 1).

### Correlations between RIPK3 level and SOFA score, CRP level, as well as PCT level

The correlations between the plasma RIPK3 level and other infection-associated biomarkers were determined. The analysis displayed that the RIPK3 level showed positive correlations with SOFA score (*r* = 0.79, *p* = 0.001), CRP level (*r* = 0.86, *p* = 0.0001), and PCT level (*r* = 0.95, *p* = 0.0001). However, the correlation between the RIPK3 level and WBC count was not significant (*r* = 0.17, *p* = 0.34). Based on the collected data, the dynamic changes in these variables indicated that the plasma RIPK3 level was a reliable predictor for patient with sepsis.

### Diagnostic accuracy of RIPK3 level, SOFA score, CRP level, and PCT level

Based on survival on day 28, patients with sepsis were divided into survivor group (*n* = 49, 39.5%) and non-survivor groups (*n* = 75, 60.5%). The RIPK3 level, SOFA score, CRP level, and PCT level were assessed by receiver operative characteristic (ROC) curve analysis in the survivor and non-survivor groups. The areas under ROC curves (AUCs) for RIPK3 level, SOFA score, CRP level, and PCT level were 0.813, 0.824, 0.756, and 0.653, respectively. These results indicated that the RIPK3 level for prognostic biomarker of sepsis was similar to that of the SOFA score (AUC, 0.813 versus 0.824; *p* = 0.659) and CRP level (AUC, 0.813 versus 0.756; *p* = 0.232). However, the RIPK3 for prognostic biomarker was significantly superior to the PCT level (AUC, 0.813 vs. 0.653; *p* = 0.013), as shown in Table 2.

**Table 2:** Areas under ROC curves (AUC) for predicting sepsis patient's prognosis

					95% confidence interval	
Variables	AUC	Cutoff value	Sensitivity	Specificity	Lower	Upper
RIPK3	0.813	14.83 pg/mL	92.15%	76.32%	0.734	0.987
SOFA	0.824	3.26	93.72%	53.21%	0.623	0.913
CRP	0.756	12.76 ng/dL	95.65%	13.21%	0.357	0.812
PCT	0.653	4.11 ng/mL	69.34%	78.56%	0.582	0.765

Abbreviations: RIPK3: receptor interacting protein kinase 3; SOFA: Sequential Organ Failure Assessment; CRP: C-reactive protein; PCT: procalcitonin.

### Prognostic value of baseline variables

Upon univariate analysis, RIPK3 level, APACHE II score, SOFA score, PCT level, CRP level, and the number of organs dysfunction were related to unfavorably short survival on day 28. However, creatinine level, WBC count, PLT count, PT, age, sex, primary infection site, microorganism identified, the type of organ dysfunction, and RIPK1 level were not correlated with fatal events (Table 3). On multivariate analysis, the RIPK3 level, APACHE II score, SOFA score, PCT level, and the number of organs dysfunction were predictor of unfavorable outcome. Unexpectedly, the CRP level was not a predictive factor in the multivariate analysis, as shown in Table 4.

**Table 3:** Univariate analysis of baseline predictors of survival patients with sepsis

Variable	Survivors (*n* = 49)	Non-survivors (*n* = 75)	*p* values
Sex (male/female)	31/18	46/29	0.85
Age (year), median (range)	60 (17–75)	61 (18–81)	0.65
APACHE II score, median (range)	18 (2–43)	26 (2–45)	0.001
SOFA score, median (range)	5.6 (0–17.5)	9.7 (0–18.2)	0.003
CRP, mg/dL, median (range)	18.43 (2.8–36.5)	24.86 (3.2–39.7)	0.001
PCT, ng/mL, median (range)	13.8 (2.1–35.24)	7.6 (3.4–36.2)	0.001
RIPK3, pg/mL, median (range)	8.9 (1.2–31.5)	17.6 (3.2–32.5)	0.001
RIPK1, pg/mL, median (range)	8.5 (1.8–17.6)	8.2 (2.1–25.1)	0.73
Creatinine, mg/dL, median (range)	1.3 (0.4–14.1)	1.2 (0.4–12.7)	0.89
WBC, × 10^9^ L, median (range)	10.9 (2.5–30.3)	11.5 (2.7–32.1)	0.18
PLT, × 10^3^ uL, median (range)	89 (56–378)	84 (29–197)	0.72
PT, seconds, mean ± SD	15.52 ± 1.47	14.95 ± 3.21	0.96
Primary infection site†			
Respiratory	33 (67.4%)	36 (48%)	0.21
Abdominal	4 (8.2%)	5 (6.7%)	
Urinary tract	5 (10.2%)	15 (20%)	
Bloodstream	1 (2.0%)	7 (9.3%)	
Others	5 (10.2%)	8 (10.7%)	
Unknown	1 (2.0%)	4 (5.3%)	
Microorganism identified			
Gram-negative bacilli	15 (30.6%)	43 (57.3%)	0.05
Gram-positive cocci	26 (53.1%)	22 (29.3%)	
Fungus	3 (6.1%)	5 (6.7%)	
Intracellular germs	2 (4.1%)	2 (2.7%)	
Others	3 (6.1%)	3 (4%)	
The kind of organ dysfunction†			
CRF	29 (52.7%)	31 (44.9%)	1.00
CHF	30 (54.5%)	35 (50.7%)	
CKD	20 (36.4%)	21 (30.4%)	
Hepatic insufficiency	9 (16.3%)	11 (15.9%)	
Immune suppression	6 (10.9%)	8 (11.6%)	
Others	2 (3.6%)	3 (4.3%)	
The number of organ dysfunction			
1	20 (40.8%)	12 (16%)	0.001
2	18 (36.7%)	25 (33.3%)	
≥ 3	11 (22.5%)	38 (50.7%)	

†Some of included patients had more than one co-morbidity.

Abbreviations: APACHE II score: Acute Physiology and Chronic Health Evaluation score; SOFA: Sequential Organ Failure Assessment; CRP: C-reactive protein; PCT: procalcitonin; RIPK3: receptor interacting protein kinase 3; RIPK1: receptor interacting protein kinase 1; WBC: white blood cell; PLT: platelet; PT: prothrombin time; CRF: chronic respiratory failure; CHF: chronic heart failure; CKD: chronic kidney dysfunction.

**Table 4:** Multivariate analysis of baseline predictors of survival of patients with sepsis

Prognostic factors	Regression coefficient (B)	Standard error	*Z* values	Significant (*p* values)	OR values	95% CI (OR)
APACHE II score	0.13	0.06	2.34	0.03	1.15	1.02–1.34
SOFA score	0.33	0.08	12.31	0.01	1.29	1.21–1.78
CRP	−0.0005	0.0005	−1.11	0.25	1.00	1.00–1.02
PCT	−0.0003	0.0002	−2.07	0.04	1.00	1.00–1.01
The number of organ dysfunction	1.13	0.56	2.05	0.04	3.09	1.05–8.96
RIPK3	0.14	0.06	4.16	0.04	1.27	1.00–1.37
cons	−3.05	1.41	−2.17	0.04	-	-

Abbreviations: APACHE II score: Acute Physiology and Chronic Health Evaluation score; SOFA: Sequential Organ Failure Assessment; CRP: C-reactive protein; PCT: procalcitonin; RIPK3: receptor interacting protein kinase 3; OR: odds ratio; CI: confidence interval.

## DISCUSSION

Increasing researchers indicate that necroptosis is playing an important role in the regulation of systemic inflammatory response syndrome and critical care medicine [12–14]. Thus, the usefulness of necroptosis regulated proteins in monitoring clinical response to treatment strategy would guide development of novel treatment strategies to patients with sepsis. The current study was to determine the prognostic biomarker of necroptosis regulated proteins in comparison to the SOFA score, PCT level, and CRP level in sepsis patients, and we found that dynamic monitoring of plasma RIPK3 levels can contribute to the prediction of outcomes of sepsis and might be of particular value in identifying patients who would benefit from specific treatments.

As always, PCT has been the most studied and promising biomarker of sepsis. Though PCT is superior to CRP and other traditional biomarkers of sepsis in critical practice, it is inaccurate for clinicians to judge clinical conditions [4–7]. Thus, it is urged to acquire much more sensitive and specific prognostic biomarkers for sepsis. In our study, the plasma RIPK3 levels in severe sepsis and septic shock patients were remarkably higher than those in sepsis patients at various time points (all *p* < 0.05), and the prognostic biomarker of the plasma RIPK3 level was comparable to that of common infection-related variables such as SOFA score and PCT level. Meanwhile, our study found that the concentration changes of plasma RIPK3 with time was similar to those of CRP levels, PCT levels, and SOFA score to monitor sepsis dynamically and then evaluate the severity of sepsis, which may be used for determining the prognosis of sepsis patients.

Moreover, among all those patients, RIPK3 levels of non-survivors were remarkably higher than those of survivors. Additionally, plasma RIPK3 levels, APACHE II score, SOFA score, PCT level, and the number of organs exhibiting dysfunction were association with a poor prognosis in sepsis patients. Given the above, we assumed that the RIPK3 levels might be as a valuable factor for determining the prognosis of sepsis.

In order to reduce the therapeutic bias, all enrolled patients received standard treatment according to the International Guidelines for Management of Severe Sepsis and Septic Shock [15]. In recent years, exciting advances have been made in the understanding of its pathophysiology and treatment strategy. Of note, between 2014 and 2015, 3 independent, multicenter, government-funded, randomized controlled trials including Protocolized Care for Early Septic Shock (ProCESS), Australasian Resuscitation in Sepsis Evaluation (ARISE), and Protocolised Management in Sepsis (ProMISe) demonstrated that early goal-directed therapy (EGDT) had shown a survival benefit [16–18].

The main limitation of this study was that the different patient populations had various causes of mortality for sepsis, which might have impacted the results. Second, the number of patients was relatively small, and they treated with heterogeneous treatment regimens. In conclusion, our results have some clinical insights that the plasma RIPK3 level may have promising value for the prognostic of sepsis, and its application could be matched by that of the SOFA score and PCT level. The plasma RIPK3 concentration is also helpful to monitor the development and prognosis of sepsis dynamically and accordingly, it has a great potential for therapeutic interventions in clinical practice. Therefore, clinical studies with larger samples sizes are further warranted to confirm the clinical value of RIP3 as a prognostic biomarker in sepsis patients.

## MATERIALS AND METHODS

### Study design

We prospectively recruited the eligible patients with sepsis, severe sepsis, or septic shock who were treated in our hospital from January 2014 to May 2016. All patients enrolled into study with written informed consent. The protocol of this study was approved by the independent ethics committees at our hospital and according to the ethical guidelines of the Declaration of Helsinki.

### Patient eligibility and exclusion

The definitions of sepsis, severe sepsis, and septic shock were in accordance with the American College of Chest Physician/Society of Critical Care Medicine (ACCP/SCCM) guidelines [19–20]. Patients suffering from cirrhosis, malignancies, diabetes, chronic organs failure, autoimmune diseases, acquired immune deficiency syndrome, or human immunodeficiency virus infection; patients who had undergone immunosuppressive, steroid, radiation, or transplantation therapies were all excluded from this study.

### Data collection and analysis

We collected peripheral blood samples from patients in different groups and then analyzed data within 24 hours start with the diagnosis of sepsis. Meanwhile, we determined the APACHE II score, SOFA score, CRP level, PCT level, creatinine level, white blood cell (WBC) count, platelet (PLT) count, prothrombin time (PT), RIPK3 level, and RIPK1 level. Besides, we recorded other clinical parameters of each patient as follow: sex, age, primary infection site, microorganism identified, the type of organ dysfunction, and the number of organs affected.

All enrolled patients received standard medical care. The routinely included antibiotics treatment, best supportive care treatment, and prevention treatment complication [15]. To evaluate outcome, the patients were followed up on day 28 after inclusion. We defined the survivor by patients who survived by the end of follow-up, yet non-survivors were patients who died within the follow-up period .

### Measurement of RIPK3 and RIPK1 level by enzyme-linked immunosorbent assay (ELISA)

The starting time point was defined from the enrolled time of those patients in the study. Venous blood plasma was collected at 24, 48, 72, and 120 hours later in a double-blinded fashion. Plasma RIPK3 and RIPK1 levels were detected with an enzyme-linked immunosorbent assay kit in line with the manufacturer's instructions (NeoBioscience Technology Co., Ltd., Shenzhen, China) as previously described [21–22].

### Statistical analysis

All quantitative data were expressed as median and range or mean ± standard deviation (SD). The nonparametric Mann-Whitney *U-test*, one-way analysis of variance, a Fisher's exact test, or Chi-squared test were used to compare the data. For the value of RIPK3, SOFA, CFR, and PCT, receiver operating characteristics curve (ROC) was plotted to calculate the area under the curve (AUC) and obtaining best cutoff values for calculating the sensitivities, specificities predictive values and likelihood ratios. A logistic regression model was used to assess the independent factors for the short-term survival rate, and the significant variables in a univariate analysis were included in a multivariate analysis. A *p*-values < 0.05 was considered to indicate statistical significance. Data were analyzed using the statistical software Intercooled Stata version 8.2 for Windows (Stata Corporation, College Station, TX, USA).
